# Serum iron status and the risk of female infertility in European populations: A two-sample Mendelian randomization study

**DOI:** 10.1097/MD.0000000000040220

**Published:** 2024-10-25

**Authors:** Ziping Liu, Zelin Zhang, Ping Xie

**Affiliations:** a Chengdu University of Traditional Chinese Medicine; b Hospital of Chengdu University of Traditional Chinese Medicine, International Ward (Gynecology), Chengdu, China.

**Keywords:** causal inference, female infertility, iron status, Mendelian randomization

## Abstract

The relationship between iron status and female infertility has been observed in several studies, yet its causal nature remains ambiguous. We employed univariate Mendelian randomization (MR) analyses to explore the potential causal connection between these 2 factors. For our analysis, genetic instrumental variables pertaining to iron status were selected using data from the Iron Status Genetics Consortium, encompassing 48,972 individuals of European descent from 19 cohorts (11 discovery and 8 replication). For female infertility data, we referred to FinnGen Consortium Release 9, which includes 11,442 cases and 107,564 controls. Our MR approach utilized both a conservative strategy (involving single nucleotide polymorphisms pertinent to general iron status) and a liberal strategy (encompassing single nucleotide polymorphisms linked to any iron status indicator). The conservative approach relied on inverse variance-weighted methods, whereas the liberal strategy integrated inverse variance weighted with MR-Egger regression, the weighted median approach, and simple mode techniques. The conservative strategy did not reveal a significant link between iron status and female infertility risk. Conversely, the liberal strategy indicated a positive correlation specifically between serum iron levels and female infertility risk (odds ratio from MR: 1.225; 95% confidence interval: 1.064–1.410; *P* = .030), while no significant associations were found for other iron indicators (*P* > 0.05). Our MR investigation suggests a potential positive association between serum iron levels and the risk of female infertility, while other iron markers do not appear to significantly influence this risk. These findings highlight the need for further research into the possible connection between serum iron status and female infertility risk.

## 1. Introduction

Infertility has become a significant public health concern worldwide. It is defined as the failure to achieve pregnancy after 12 months of regular, unprotected intercourse, or within 6 months for women over 35 years old. This condition affects around 15% of couples of childbearing age.^[1–3]^ The global lifetime risk of infertility varies, with estimates ranging from 2.6% to 31.8%,^[4,5]^ although these numbers might be underestimated.^[6]^ In China, infertility rates in the reproductive population are as high as 25%. Notably, almost half of those affected do not seek medical intervention, underscoring the gravity of the situation.^[7,8]^

Iron, an essential micronutrient predominantly found in meats, seafood, fortified grains, legumes, and spinach, is crucial for various bodily functions. It is integral to hemoglobin, cytochromes, and myoglobin, playing roles in oxygen transport, DNA replication, and adenosine triphosphate production. Furthermore, iron is implicated in disturbances in glucose and androgen metabolism,^[8–10]^ as well as compromised immune function,^[11,12]^ which could potentially affect fertility.

The link between iron status and female infertility has attracted considerable attention in recent times. Epidemiological studies have observed that women with unexplained infertility often have lower transferrin saturation and mean corpuscular hemoglobin.^[13]^ Profound iron deficiency is thought to be a significant factor in unexplained infertility cases.^[14]^ However, a study by Skalnaya et al^[15]^ found no notable difference in serum iron levels between healthy individuals and pregnant women, but a 13% increase in women who experienced miscarriages compared to those who did not. Another research indicated lower serum ferritin levels and a heightened risk of iron deficiency in women with recurrent miscarriages.^[16]^ In epidemiological and genetic studies, exposure refers to factors or conditions that may increase or decrease the probability of occurrence of a specific health outcome.^[17]^ When investigating female infertility, exposure may encompass environmental factors (such as lifestyle, exposure to chemical substances),^[18]^ biological factors (including hormonal levels, reproductive system disorders),^[19]^ among others. Although current observational studies suggest a potential direct causal relationship between iron status (as an exposure factor) and infertility, the confirmation of this relationship is often confounded by various factors (e.g., age, socioeconomic status), making it challenging for traditional observational studies to accurately assess the association. Therefore, more rigorous methodologies are necessary to validate this potential causal link.

Mendelian randomization (MR), an epidemiological technique, is employed to ascertain the causal relationships between exposures and outcomes using genetic variants as instrumental variables.^[20]^ Adhering to Mendel’s laws of inheritance, MR maintains the randomness and independence of genetic data, free from the influence of external environmental and behavioral factors.^[21]^ MR is particularly advantageous over traditional observational studies in mitigating confounding issues and reversing causality. Given the constraints of randomized controlled trials in examining the impact of iron status on female infertility, the role of MR analysis becomes crucial. In this study, we postulate a causal link between iron status and female infertility and undertake a univariate MR analysis to test this hypothesis.

## 2. Materials and methods

### 2.1. Study design and data sources

**Figure 1.**
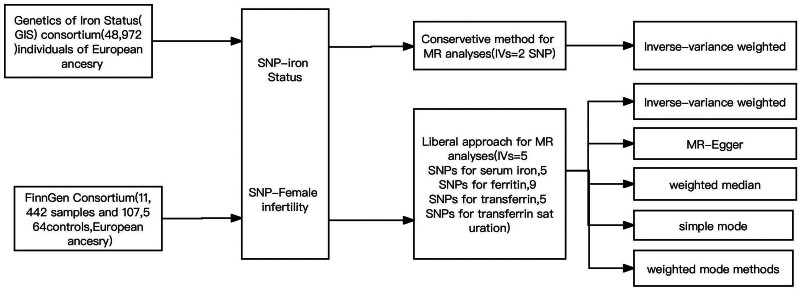
Overview of databases and analytical strategies in Mendelian randomization analysis. This figure provides a schematic representation of the data sources and analytical methodologies utilized in our MR analysis. We extracted public summary data on SNP phenotypes from the most comprehensive meta-analysis GWAS database available. Our study adopted both conservative and liberal approaches in assessing the impact of iron status on female infertility. The conservative approach was more stringent, selecting IVs only if they were associated with an overall increase in iron status. This includes increased ferritin concentration, serum iron, and transferrin saturation, coupled with a decreased transferrin concentration. In contrast, the liberal approach was broader, incorporating any SNP associated with female infertility. For the estimation process, the conservative method exclusively used the IVW method. The liberal method, however, employed a combination of techniques: IVW, MR-Egger regression, the weighted median approach, and the simple mode method. GWAS = genome-wide association studies, IV = instrumental variable, IVW = inverse variance weighted, MR = Mendelian randomization, MR-Egger = Mendelian Randomization-Egger regression method, SNP = single nucleotide polymorphism.

Our study employed a 2-sample MR framework, integrating data from diverse genome-wide association studies (GWAS) as outlined in Table 1 and illustrated in Figure 1. The foundational studies for these GWAS databases were conducted under strict adherence to ethical standards, including obtaining informed consent from all participants and necessary ethical approvals.

**Table 1:** Data sources.

Exposure/outcome	Trait	Consortium/cohort study	Ethnicity	Subject	Pubmed ID or web source
Novel loci affecting iron homeostasis and their effects in individuals at risk for hemochromatosis	Serum iron, transferrin, transferrin saturation, and ferritin	Genetics of Iron Status	European	48,972 subjects	PMID: 36145059
Genetic instruments for female infertility	Female infertility	FinnGen Consortium	European	11,442 samples and 107,564controls	(https://r9.finngen.fi/, accessed on November 24, 2023)

In our analysis, single nucleotide polymorphisms (SNPs) strongly linked to total serum iron levels were utilized as instrumental variables. This approach allowed us to examine the influence of iron status on female infertility risk. We adjusted the associations between these SNPs, iron status, and female infertility to estimate the overall impact of iron status on infertility risk. Additionally, we delved into the connections between iron status and other known risk factors for female infertility. This was aimed at determining whether iron status could potentially act as a biasing factor or a mediator in the context of female infertility.

**Figure 2.**
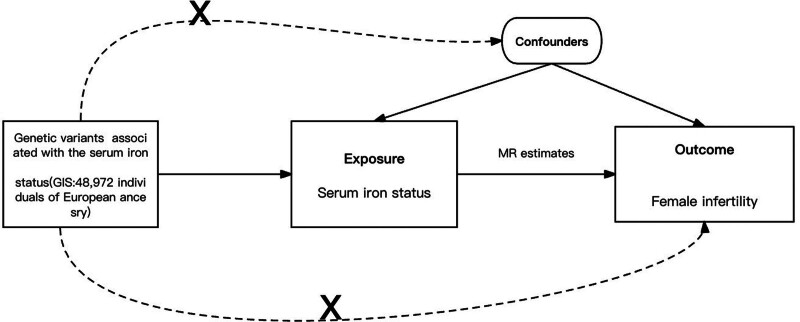
Diagram view of the 2-sample MR study design. MR = Mendelian randomization, SNP = single nucleotide polymorphism.

Figure 2 depicts our MR study design, based on 3 core assumptions: Association with iron-related traits: IVs are specifically chosen for their direct links to serum iron, transferrin, ferritin, and transferrin saturation; Confounder independence: These IVs are selected for their independence from known confounders, ensuring that observed relationships are unbiased; Direct pathway to female infertility: The IVs are assumed to influence female infertility solely through their impact on serum iron-related traits, excluding alternative pathways. This design underpins our approach to establish a clear causal relationship between iron status and female infertility.

The genetic data regarding serum iron status in this study were derived from a GWAS of 48,972 individuals of European descent.^[22]^ This GWAS provided summary data on genome-wide allele associations with iron markers from 23,986 participants across 11 cohorts from 9 research centers. Additional replication samples came from 24,986 participants in 8 cohorts. Genome-wide association tests, along with genotype imputation and quality control, were independently executed in each cohort.^[14]^ The analysis of genotype-iron phenotype associations utilized an additive model, adjusted for age, gender, principal component scores, and other covariates specific to each study. This GWAS was approved by appropriate review committees, and its methodologies have been published in detail.^[22]^

For female infertility genetic variants, we referred to the FinnGen consortium’s GWAS (accessed on November 24, 2023: [https://r9.finngen.fi/]). From this, using R9 version data based on International Classification of Diseases 9 and International Classification of Diseases 10 codes and excluding specific genders, we identified 11,442 cases and 107,564 controls. As our study employed published summary statistics, no further ethical approval was required.

### 2.2. Selection of instrumental variables

We selected SNPs closely associated with iron status as instrumental variables from the Genetics of Iron Status Consortium (*P* < 5 × 10^−8^), ensuring they had no significant link with female infertility (*P* > .05). To account for confounders like body mass index and age at menarche, which are related to infertility risk,^[23]^ we used the PhenoScanner V2 website (accessed on December 17, 2023: [http://www.phenoscanner.medschl.cam.ac.uk/]) to exclude SNPs associated with these confounders or infertility, thereby eliminating pleiotropy. Linkage disequilibrium analysis was performed to ensure SNP independence (*r*^2^ > 0.001). Specifics of the genetic instruments used in our univariate MR analysis are in Supplementary Tables S1–S4, Supplemental Digital Content, http://links.lww.com/MD/N783.

Two SNP selection strategies were employed: conservative and liberal.^[24,25]^ The conservative approach selected 3 SNPs (rs855791, rs1800562, rs1799945) for their strong links with improved serum iron status (increased iron and ferritin levels, increased transferrin saturation, decreased transferrin levels, *P* < 5 × 10^−8^). However, due to its palindromic nature, rs1799945 was excluded from this analysis, leaving rs855791 and rs1800562. The liberal approach involved selecting SNPs from GWAS closely linked with each iron status marker (*P* < 5 × 10^−8^): 4 SNPs for serum iron (rs8177240, rs1800562, rs7385804, rs855791), 5 SNPs for ferritin (rs1800562, rs411988, rs855791, rs744653, rs651007), 6 SNPs for transferrin (rs744653, rs8177240, rs1800562, rs4921915, rs6486121, rs855791), and 5 SNPs for saturation (rs8177240, rs1800562, rs7385804, rs855791, rs651007). SNPs rs9990333 and rs1799945 were excluded in the liberal approach due to incompatible alleles and palindromic nature, respectively. Descriptive statistics for these SNPs can be found in previously published studies.^[24,25]^

### 2.3. Validation of selected instrumental variables

To confirm the suitability of the selected SNPs as instrumental variables, we adhered to 3 stringent criteria.^[26]^ Initially, the SNPs must demonstrate a strong association with serum iron status. Secondly, it is imperative that these SNPs have no connections to any confounders that might affect the serum iron status and female infertility relationship. Thirdly, the SNPs should influence female infertility exclusively through their impact on serum iron status. To mitigate the risk of weak instrumental variable bias, we ensured robustness by confirming that the *F* statistics for our instrumental variables were consistently above the threshold of 10.^[27]^ This consideration is crucial for the reliability of our study. Additionally, to diminish bias arising from population stratification, both the exposure and outcome groups were comprised solely of individuals of European descent.

We undertook 3 specific analyses to address potential pleiotropic effects. The first analysis involved scrutinizing the SNPs for associations with known risk factors, such as body mass index and female age at menarche. Next, we applied 2 distinct methodologies—conservative and liberal—to our MR estimates. Lastly, we utilized the MR-Egger test to evaluate the presence of unknown directional pleiotropy.

### 2.4. MR analysis

In this study, we employed a 2-sample MR approach to explore the causal link between iron status and female infertility. Our analysis was facilitated using the “TwoSampleMR” R package (version 0.5.6), developed by Stephen Burgess in Chicago, Illinois, USA. The primary method for our analysis was the inverse variance weighted (IVW; random effects) approach, supplemented by several other methods: MR-Egger regression, weighted median, simple mode, and weighted mode. The IVW method is highly effective when all SNPs function as valid instrumental variables, providing accurate estimates.^[28]^ MR-Egger regression is beneficial for identifying and adjusting for pleiotropy but tends to be less precise.^[29]^ The weighted median method, assuming that a majority (at least 50%) of the instrumental variables are valid, is known for its accuracy.^[30]^ While the simple mode method might have lower power compared to IVW, it offers greater robustness against pleiotropy.^[31]^ Finally, the weighted mode is a method that requires careful bandwidth selection for accurate model estimation.^[32]^

### 2.5. Sensitivity analysis

In our sensitivity analysis, we utilized the IVW method and MR-Egger tests to assess heterogeneity, with forest plots employed to illustrate the contribution of each SNP, accompanied by Cochran’s *Q* statistics.^[33,34]^ Additionally, a leave-one-out approach was adopted, where each SNP was sequentially excluded from the analysis to recalculate the estimates of the remaining instrumental variables. This method helped identify any SNP exerting a disproportionately large or influential effect. To further strengthen the MR analysis, an MR-Egger statistical sensitivity analysis was conducted to mitigate potential pleiotropic effects of the instrumental variables. In this analysis, the MR-Egger regression’s intercept is used as a measure of average pleiotropic bias, which is estimated independently.^[35]^ However, the conservative set of SNPs was excluded from this sensitivity analysis due to its small size of only 2 SNPs, which could compromise the reliability of the inferences. Furthermore, no MR-Egger regression for pleiotropy testing was applied to the conservative approach, as the limited data from just 2 SNPs was insufficient.^[36]^ This limitation precluded the evaluation of potential pleiotropic effects between iron and transferrin saturation on the overall risk of female infertility.

### 2.6. Further validation of MR findings

To reinforce the credibility of our MR results, additional analyses were conducted utilizing maximum likelihood, penalized weighted median, and IVW with fixed effects methods.

The maximum likelihood method, a conventional approach in statistical analysis, is characterized by its lower standard errors. It functions by estimating parameters of the probability distribution that maximize the likelihood function.^[37]^ While this method can incur small biases due to limited sample sizes, these biases are generally considered biologically insignificant.^[38]^

The IVW method with fixed effects is particularly effective when all instrumental variables are valid. However, it is notably sensitive to invalid IVs.^[39]^ This sensitivity makes it a valuable tool for evaluating the validity of the instrumental variables used in the study.

## 3. Results

### 3.1. MR analysis of genetic instruments for serum iron status

In our MR analysis, the conservative approach utilized 2 SNPs that were associated with all 4 primary biomarkers of serum iron status. Conversely, the liberal approach incorporated a broader range of SNPs: 4 significantly linked to serum iron, 5 each to ferritin and saturation, and 6 to transferrin.

Crucially, all selected SNPs in both approaches exhibited *F* statistics greater than 10 (with a range from a minimum of 40 to a maximum of 2947), thus effectively circumventing any issues related to weak instrumental variable bias.^[39]^ This robustness in *F* statistics lends greater credibility to the instrumental variables and, by extension, to the findings of the MR analysis.

### 3.2. Effect of iron status on female infertility

**Figure 3.**
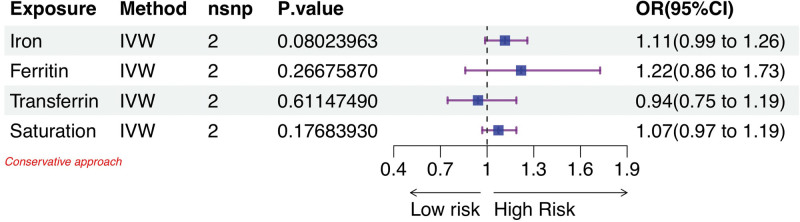
MR estimates the causal effect between indicators of 4 iron status and female infertility under conservative analytical methods. CI = confidence interval, IVW = inverse variance weighted, MR = Mendelian randomization, OR = odds ratio.

**Figure 4.**
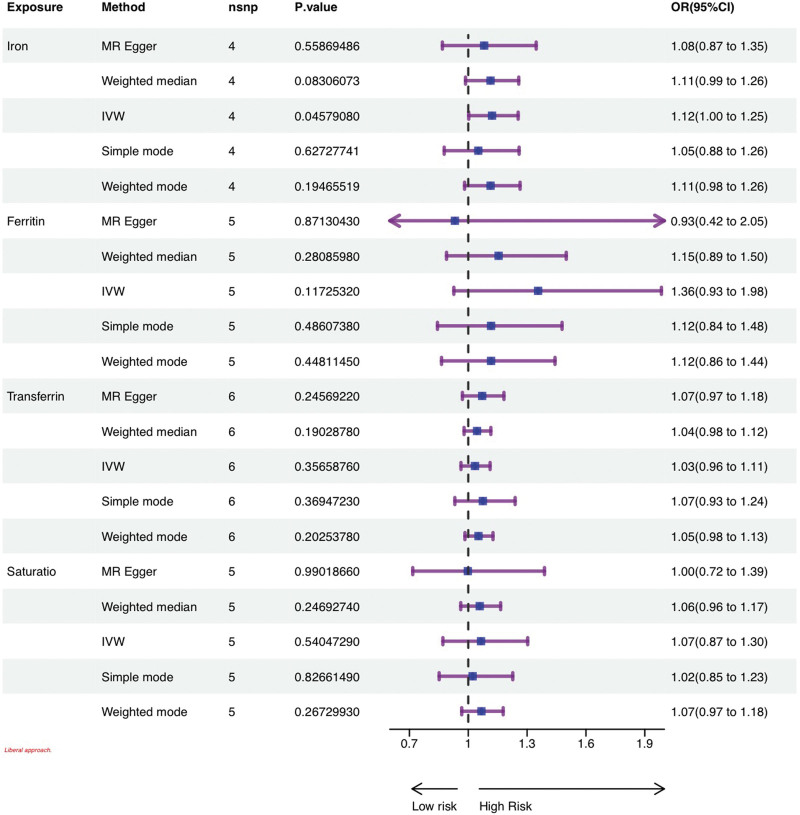
Estimation of causal effects between indicators of 4 iron status and female infertility by MR under a libertarian analytical approach. MR = Mendelian randomization.

Figure 3 illustrates the outcomes of the conservative MR analysis, which investigated the correlation between genetically predicted iron status and female infertility risk. It displays the odds ratios (ORs) for female infertility per standard deviation increase in each iron marker level. The conservative analysis revealed no significant associations between any of the 4 iron status markers and the risk of female infertility (all *P* > .05). Conversely, Figure 4 presents the results from the liberal approach. Here, the IVW analysis identified a positive correlation specifically between serum iron levels and the risk of female infertility. The OR was determined to be 1.12 (95% confidence interval: 1.00–1.25; *P* = .0457), suggesting an 11% increase in infertility risk for each 1 mmol/L elevation in serum iron levels. However, similar to the conservative approach, this analysis found no significant relationships between other iron markers and the risk of female infertility (*P* > .05). Specific values for this analysis can be found in Supplementary Tables S5 and S6, Supplemental Digital Content, http://links.lww.com/MD/N783.

### 3.3. Sensitivity analysis

Sensitivity analyses were performed to assess the stability and reliability of the IVW findings. The heterogeneity assessments using both IVW and MR-Egger methods (as presented in Supplementary Table S7, Supplemental Digital Content, http://links.lww.com/MD/N783) revealed no significant heterogeneity in the analysis exploring the link between serum iron concentration and the risk of female infertility (*P* > .05). Additionally, the MR-Egger regression outcomes (detailed in Supplementary Table S8, Supplemental Digital Content, http://links.lww.com/MD/N783) suggested the absence of pleiotropy in the MR results (*P* > .05).

Crucially, the MR estimates proved consistent across the leave-one-out analysis, which indicates the robustness of the results. Despite minor variations in the direction of estimation, the overall conclusions remained unaffected (as depicted in Supplementary Figures 1–3, Supplemental Digital Content, http://links.lww.com/MD/N782). This demonstrates the findings’ resilience to individual SNP variations and reinforces the integrity of the MR analysis.

### 3.4. Further validation of MR findings

The leave-one-out SNP analysis highlighted specific SNPs potentially impacting the IVW results, with some outcomes diverging from previous epidemiological studies. To validate these findings further, we expanded our analysis beyond the 5 traditional methods to include maximum likelihood, penalized weighted median, and IVW (fixed effects) approaches. The analyses through IVW (fixed effects) and penalized weighted median methods (as detailed in Supplementary Table S9, Supplemental Digital Content, http://links.lww.com/MD/N783) both underscored a positive link between serum iron concentration and female infertility risk, with an OR of 1.12 (95% confidence interval = 1.00–1.25, *P* = .045). This additional validation solidifies the observed positive association between elevated serum iron levels and an increased risk of female infertility.

## 4. Discussion

Prior observational studies have yielded inconsistent results on the link between iron status and female infertility, likely due to differences in study populations, including racial, ethnic, and sample size variations. The possibility of reverse causation and overlooked confounding factors could also skew the observed associations. Addressing these challenges, our research leveraged extensive GWAS summary data from European cohorts and employed a 2-sample MR approach to explore the causal relationship between iron status and female infertility risk. The conservative MR analysis found no significant association between iron status and infertility risk. However, liberal analysis revealed a positive correlation between serum iron levels and increased risk of infertility, while no such link was established for other iron status indicators.

With the advancement of eugenics, women in the preconception period are paying more attention to healthy diets and adequate nutrient intake.^[40]^ Iron, an essential nutrient, is involved in various cellular functions such as red blood cell function, oxygen transport, DNA synthesis, protein synthesis, hormone synthesis, and cell replication. Iron deficiency is commonly associated with anemia, neurodegenerative diseases, growth retardation, and behavioral disorders.^[41,42]^ Some studies suggest that iron deficiency might lead to infertility or recurrent miscarriage,^[43]^ but the findings on the impact of iron supplementation on female pregnancy are inconsistent. One study revealed that iron supplementation during the preconception period was associated with an increased pregnancy rate among women; however, the research was limited by its small sample size, involving only 8 participants.^[44]^ Subsequently, a comprehensive review identified an extensive 8-year prospective study involving 18,555 premenopausal women. This study demonstrated a significant decrease in infertility odds among women who consistently took iron supplements compared to those who did not.^[45]^ In contrast, an analysis combining data from 2 prospective cohort studies, conducted in Denmark and North America between 2013 and 2018, reported a minimal correlation between dietary heme iron intake and female fertility.^[40]^ Thus, to further clarify the relationship between serum iron levels and female infertility, our study employed a MR approach.

In our investigation, the conservative MR analysis found no significant link between serum iron status and female infertility, aligning with some prior research outcomes. Conversely, the liberal analysis revealed a positive association, suggesting that elevated serum iron levels correlate with an increased risk of infertility. This result, diverging from certain earlier epidemiological findings, prompted further validation through not only traditional methods but also advanced techniques like maximum likelihood, penalized weighted median, and IVW (fixed effects) methods. Both the IVW (fixed effects) and penalized weighted median approaches confirmed this positive correlation (OR = 1.12 [1.00–1.25], *P* = .045). Aware of the potential for pleiotropy in MR analysis,^[46]^ we conducted a comprehensive search for relevant SNPs in the PubMed database. This led to the identification of rs1800562 locus in the *HFE* gene, known to influence TG levels and purportedly affect female infertility risk.^[23]^ Yet, removing this SNP from our liberal analysis sustained the positive association between serum iron levels and infertility risk (OR = 1.15 [1.008–1.317], *P* = .037), suggesting lipid levels’ minimal impact on our findings.

Sensitivity analysis revealed no significant differences between the conservative and liberal approaches. Variations in estimates or confidence intervals across MR methods are likely attributable to random variability or measurement errors rather than actual discrepancies. Additionally, the MR-Egger method found no evidence of pleiotropy bias. Utilizing public GWAS data from European cohorts minimized population bias. Our leave-one-out MR analysis, aligning with the primary MR findings, suggests our results and conclusions are robust and largely unbiased.

Iron is vital for life, involved in numerous cellular functions. However, excess iron poses risks due to its oxidative properties.^[47]^ The Fenton reaction (Fe_2_^+^ + H_2_O_2_ → Fe_3_^+^) transforms hydrogen peroxide into free radicals, damaging tissues. Given the body’s limited iron storage and excretion capacity, overconsumption of iron-rich foods may lead to tissue iron accumulation and formation of reactive iron complexes.^[7]^ Studies have shown that small dietary iron amounts (about 5 µg, equivalent to 0.05% ferrous ions) can disrupt lipid and carbohydrate metabolism, causing embryotoxicity.^[38]^ Wang and Pantopoulos research highlights iron’s role as a stress-induced intracellular messenger, modulating toxicity and cell death in various conditions.^[48]^ Iron’s involvement in cellular redox balance is critical, and its dysregulation can cause cell and tissue damage, potentially increasing early miscarriage risk.^[15,49]^ Recent findings suggest iron overload in conditions like endometriosis may lead to embryotoxicity and ferroptosis, contributing to infertility.^[23]^ Elevated circulating hepcidin levels in infertile women also imply a crucial link between iron homeostasis and infertility.^[50]^ The intricate link between iron status and female infertility’s pathogenesis underscores the elusive nature of their causal relationship. Disruption in iron homeostasis leads to continuous redox reactions, releasing numerous free radicals, such as superoxide anions, hydroxyl radicals, and hydrogen peroxide.^[51]^ Excess iron, by facilitating reactive oxygen species production, may adversely impact ovarian follicle formation.^[52]^ Thus, investigating the iron-infertility nexus is both complex and essential.

In summary, it is difficult to say which is the exposure and which is the outcome in previous studies. Our study leverages a substantial European dataset to conduct MR analysis, clarifying the causal relationship between iron status and female infertility. We found a positive association between serum iron levels and infertility risk, while other iron markers showed no significant link. To our knowledge, this is the inaugural study employing MR to investigate this relationship. However, the study is not without its limitations. Primarily, it focused on individuals of European descent, limiting the generalizability of the findings to other populations. Confounding variables such as age, gender, and environmental factors also influenced the MR analysis outcomes. Additionally, the use of different assay kits for measuring serum iron, ferritin, transferrin saturation, and transferrin introduced potential systematic errors. Future research should aim for consistency in assay methods to minimize discrepancies. Moreover, our analysis is constrained by the scarcity of comparative studies on serum iron parameters and female infertility, limiting our interpretation to the direct causal relationship between iron status (as the exposure) and female infertility (as the outcome). Despite these limitations, our study sheds light on the genetic underpinnings of the relationship between iron homeostasis and female infertility, offering valuable insights for subsequent research in this field.

## Acknowledgments

We would like to express our gratitude to the participants and researchers of the FinnGen study and the Iron Status Genetics Consortium.

## Author contributions

**Conceptualization:** Ziping Liu.

**Data curation:** Ziping Liu.

**Writing—original draft:** Ziping Liu.

**Investigation:** Zelin Zhang.

**Writing—review & editing:** Ping Xie.

## Supplementary Material


