# Supplementary material for: Serum iron status and the risk of female infertility in European populations: A two-sample Mendelian randomization study
Source: Medicine (Baltimore). 2024 Oct 25;103(43):e40220. doi: 10.1097/MD.0000000000040220 (PMC11521090; doi:10.1097/MD.0000000000040220)
Supplement: Supplementary file 2 [file medi-103-e40220-s002.pdf]

Supplementary Figure S1

Figure S1. Scatter plot of MR effect size for causal associations between GIS Serum Iron Status and female infertility.(A:Iron and female infertility；B:Ferritin and female infertility；C:Transferrin and female infertility；D:Saturation and female infertility）

Figure S1. Scatter plot of MR effect size for causal associations between GIS Serum Iron Status and female infertility.

A：Iron and female infertility

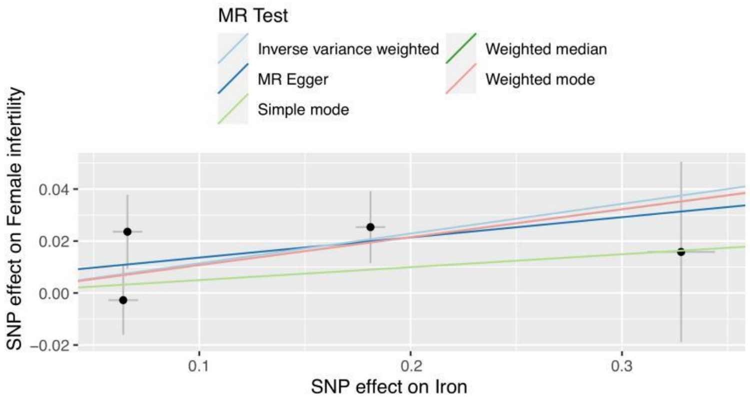

B:Ferritin and female infertility

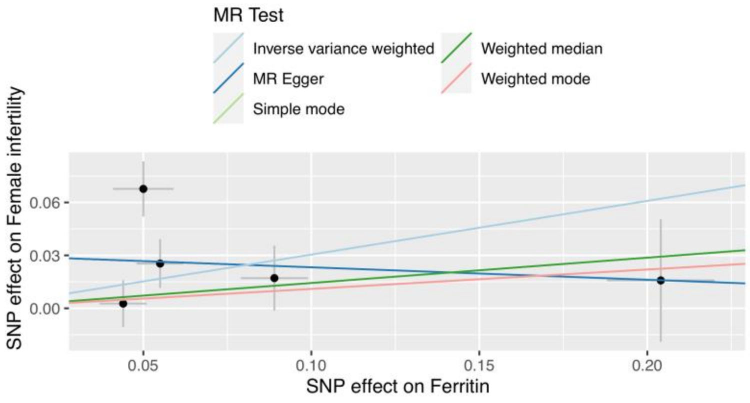

C:Transferrin and female infertility

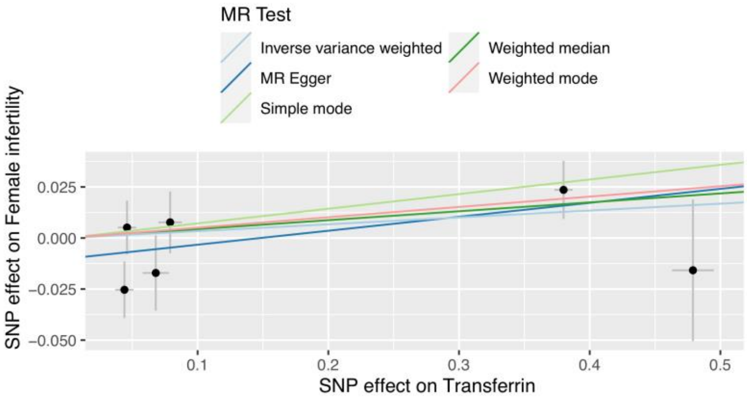

D:Saturation and female infertility

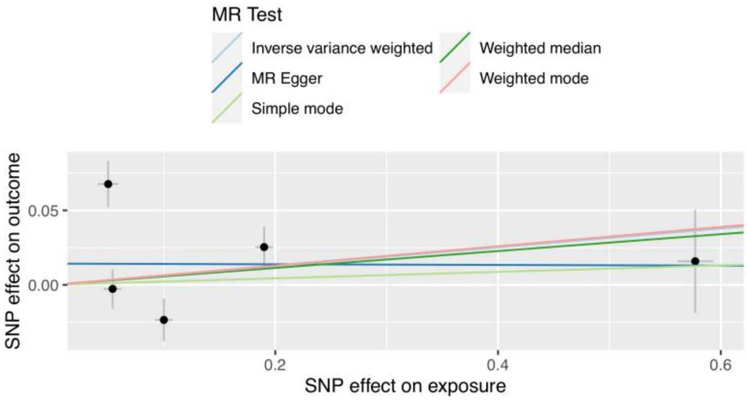

Supplementary Figure S2

Forest plot of MR effect size using MR-Egger and IVW methods for causal associations between GIS Serum Iron Status and female infertility. (A:Iron and female infertility；B:Ferritin and female infertility；C:Transferrin and female infertility；D:Saturation and female infertility）

A:Iron and female infertility

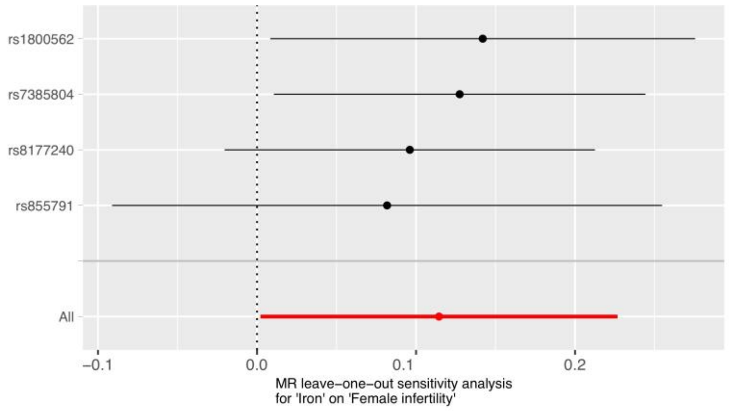

B:Ferritin and infertility

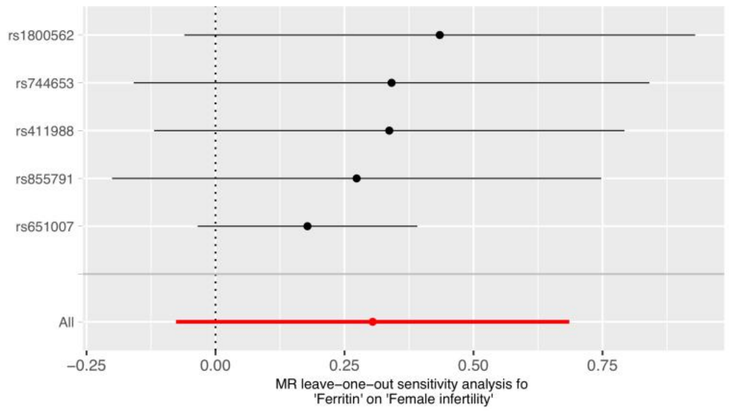

C:Transferrin and infertility

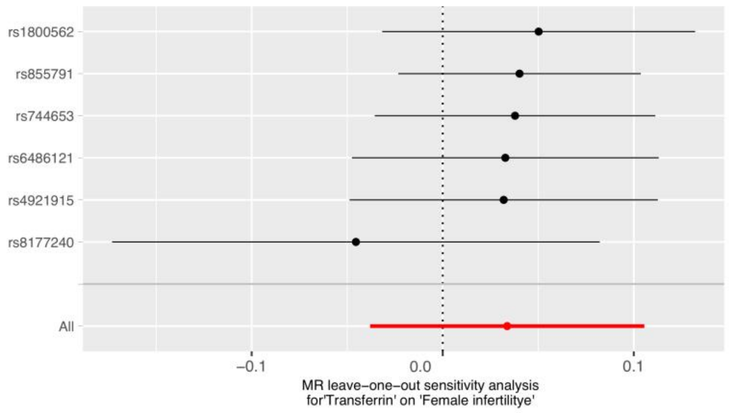

D:Saturation and female infertility

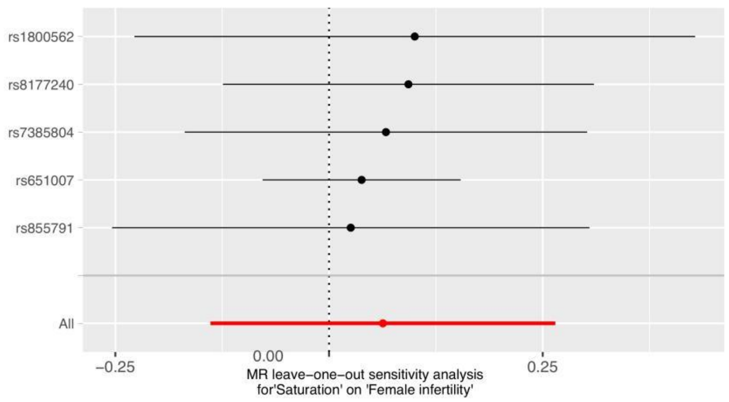
rs1800562
rs8177240
rs7385804
rs651007
rs855791
All
−0.25
0.00
0.25
MR leave−one−out sensitivity analysis
for'Saturation' on 'Female infertility'

Supplementary Figure S3

Funnel plot of causal associations between GIS Serum Iron Status and female infertility. (A:Iron and female infertility；B:Ferritin and female infertility；C:Transferrin and female infertility；D:Saturation and female infertility).

A：Iron and female infertility

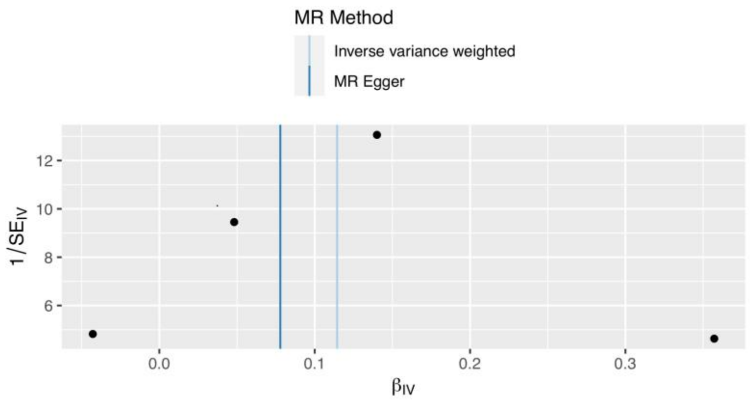

B:Ferritin and female infertility

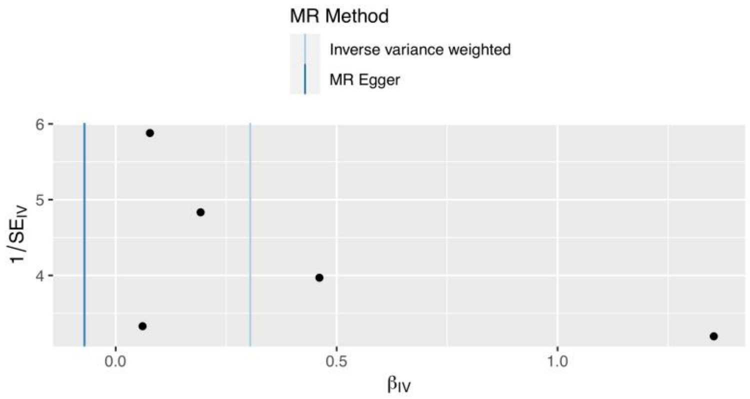

C:Transferrin and female infertility

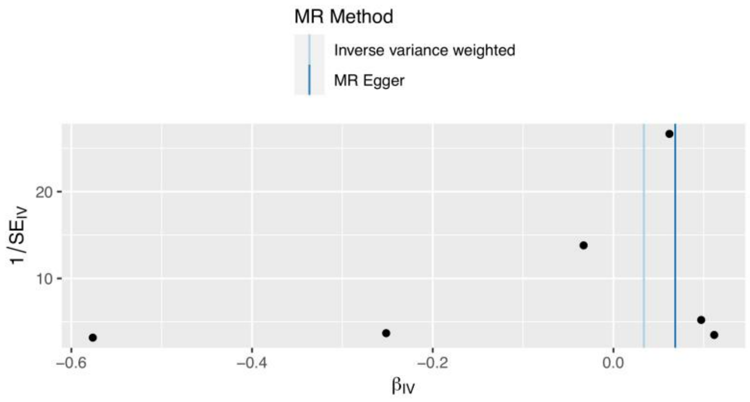

D:Saturation and female infertility

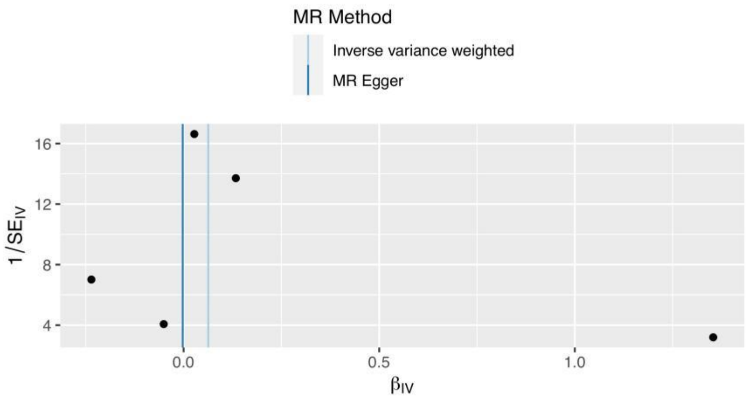
MR Method
Inverse variance weighted
MR Egger
16
12
8
4
1/SE$_{IV}$
0.0
0.5
1.0
β$_{IV}$
